# Supplementary material for: Control of synchronization ratios in clock/cell cycle coupling by growth factors and glucocorticoids
Source: R Soc Open Sci. 2020 Feb 12;7(2):192054. doi: 10.1098/rsos.192054 (PMC7062057; doi:10.1098/rsos.192054)
Supplement: Supplementary Material [file rsos192054supp1.pdf]

Supplementary Material

Control of Synchronization Ratios in Clock/Cell Cycle Coupling by Growth Factors and Glucocorticoids

S. Almeida, M. Chaves, F. Delaunay

1 Period Measurement Methods

Computing the period of a system in a biological context poses particular issues. In a system with a complex periodic behavior, different levels of protein peak expression may appear and it is fundamental to identify which ones would have biological meaning.

Using the mathematical period T of the solution, $x(t) = x(t+T)$ leads to the rejection of peaks of protein expression that may be relevant in a biological context. For example, if the clock has a complex behavior, peaks of protein expression occuring within the time interval of one mathematical period could in the real system indicate a detectable clock oscillation if high enough relatively to the protein's maximum value. Similarly, in a bidirectional coupling configuration, where MPF may also exhibit complex behavior, a peak of MPF that is slightly below the maximum may be sufficient to carry out the mitotic process, while peaks bellow a certain threshold have no effect in mitosis, which is a discrete process. Thus, it is necessary to select a threshold for the selection of relevant protein peaks.

Our algorithm is based on a numerical implementation of the first return map [1]. We consider for each protein a threshold of 50 % of their maximum value and compute the time differences between the points of the two Poincaré sections crossing the threshold (one section containing points where the signal is increasing and the other the points where the signal is decreasing), and average them. This method considers only the peaks that are above the threshold and ignores small amplitude peaks of our numerical solutions, that wouldn't be distinguishable in experimental results. Thus, in some cases of complex behavior, this period will not correspond to the mathematical period T of the solution, but will nevertheless provide a more realistic estimate of the period-lock ratios. This approach allows to better relate our work with experimental observations of clock to cell cycle period ratios observed in mammalian cells [2]. In fact, in biology normal variation among protein peaks is to be expected.

In Fig. 1 the simulation shown in Fig. 3 of the main article (bottom panel) is repeated for the thresholds of 30 and 99 % of the maximum value. One main difference between Fig. 3 and Fig. 1 A) is that the shifts between synchronization states occur for different values of GF. Nevertheless, the same values of r_T appear. Moreover, Fig. 1 B) shows the synchronization state for a very high protein threshold (99 % of the maximum value), which results in computing the mathematical period, where the 6:1 ratio occurs.

Furthermore, Fig. 2 gives an example of a solution of unidirectional cell cycle $\rightarrow$ clock coupling, where the clock has a complex behavior: observe that a complete clock period occurs during the time inter-

val of 6 cell cycle periods, however there are several intermediary peaks of BMAL1 and REV that are high enough relatively to their maximum value and could in the real system indicate a detectable clock oscillation. In this simulation, using either a 30 % or 50 % threshold to define the relevance of peaks allows to detect 4 peaks of BMAL1 to 6 of MPF (3:2 period-lock), but using, for instance, a 75 % threshold, would only count 3 peaks of BMAL1 as relevant in the time interval of 6 MPF oscillations (2:1 period-lock). The 99 % threshold measures the mathematical period, where a repetition of the higher peak of BMAL1 occurs for 6 peaks of MPF (6:1 period-lock) and all remaining BMAL1 peaks are ignored.

Because coupling from the cell cycle is being modeled as an action on REV, this protein tends to have a more complex behavior than the remaining clock proteins – in Fig. 2 using a threshold of 75 % would count 3 peaks of BMAL1 and only 2 of REV in the time interval of 6 MPF periods, while the 30 % and 50 % thresholds allow to count 4 peaks of each of these proteins in the same time interval. In this work, we base our computations of period-lock ratios on BMAL1 to avoid possible errors. However, using the chosen threshold (50 %) of mean protein value for each protein tends to yield similar results between REV and the other proteins.

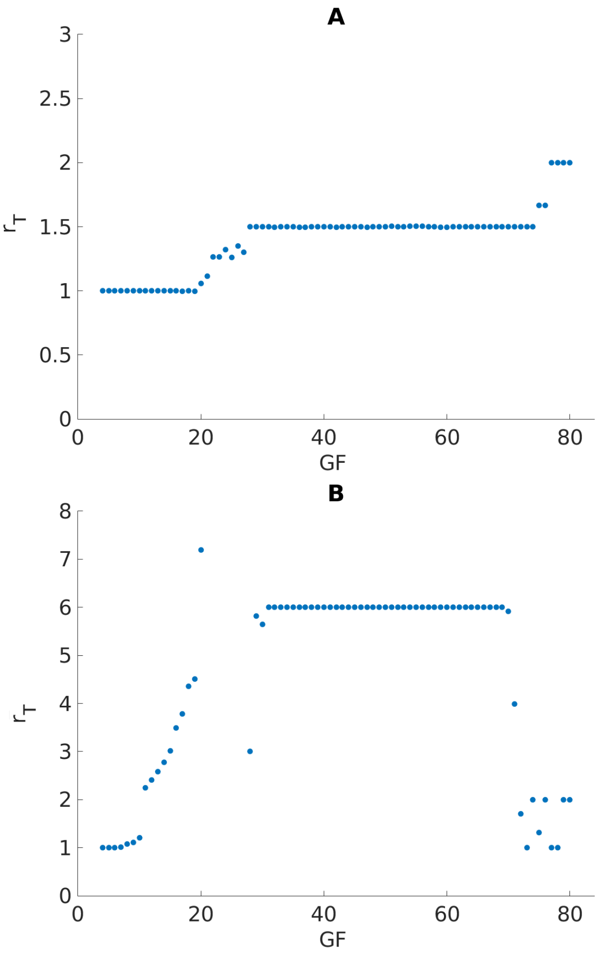

Fig. 1: **Synchronization ratios for different period measurement thresholds in unidirectional cell cycle → clock coupling with $\mathbf{c_m = 0.08}$.** Same simulation of Fig. 3 (main text, bottom panel) using different thresholds of peak count. A) Threshold of 30 % of the maximum protein value alters slightly the GF points of transition between synchronization ratios. B) With a threshold of 99 % of the maximum we are measuring the mathematical period, presenting a 6:1 clock to cell cycle period-lock.

2 Supporting Figures

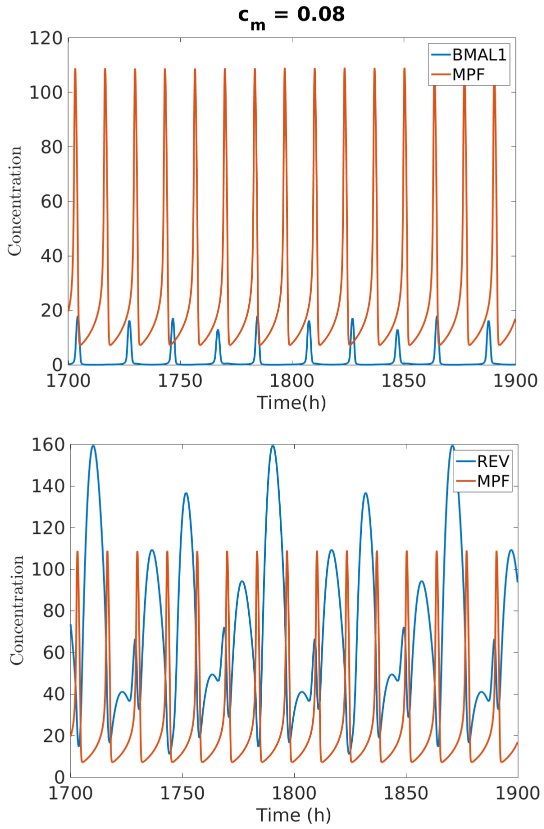

Fig. 2: **Oscillations of BMAL1 and MPF in a 3:2 period-lock.** With GF = 40 and coupling strength $c_m = 0.08$ the solution shows a 3:2 period-lock, where for four peaks of BMAL1, there are six peaks of MPF. There are also two relevant peaks of REV (above mean REV value) for each three relevant peaks of MPF (above mean MPF value). The cell cycle period is kept constant at 14.5 h in the unidirectional coupling, the clock period (computed as the average of the time difference between peaks of BMAL1) is 21.7 h.

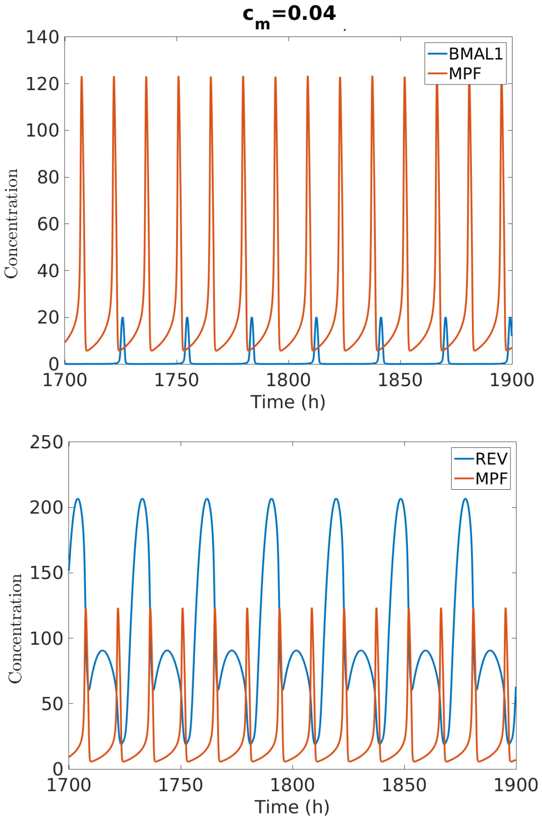

Fig. 3: **Oscillations of BMAL1, REV and MPF in a 2:1 period-lock.** While keeping GF = 40, the cell cycle period is kept constant at 14.5 h in the unidirectional coupling. The coupling strength $c_m = 0.04$ results in a solution with a 2:1 period-lock, where the clock period is 28.9 h.

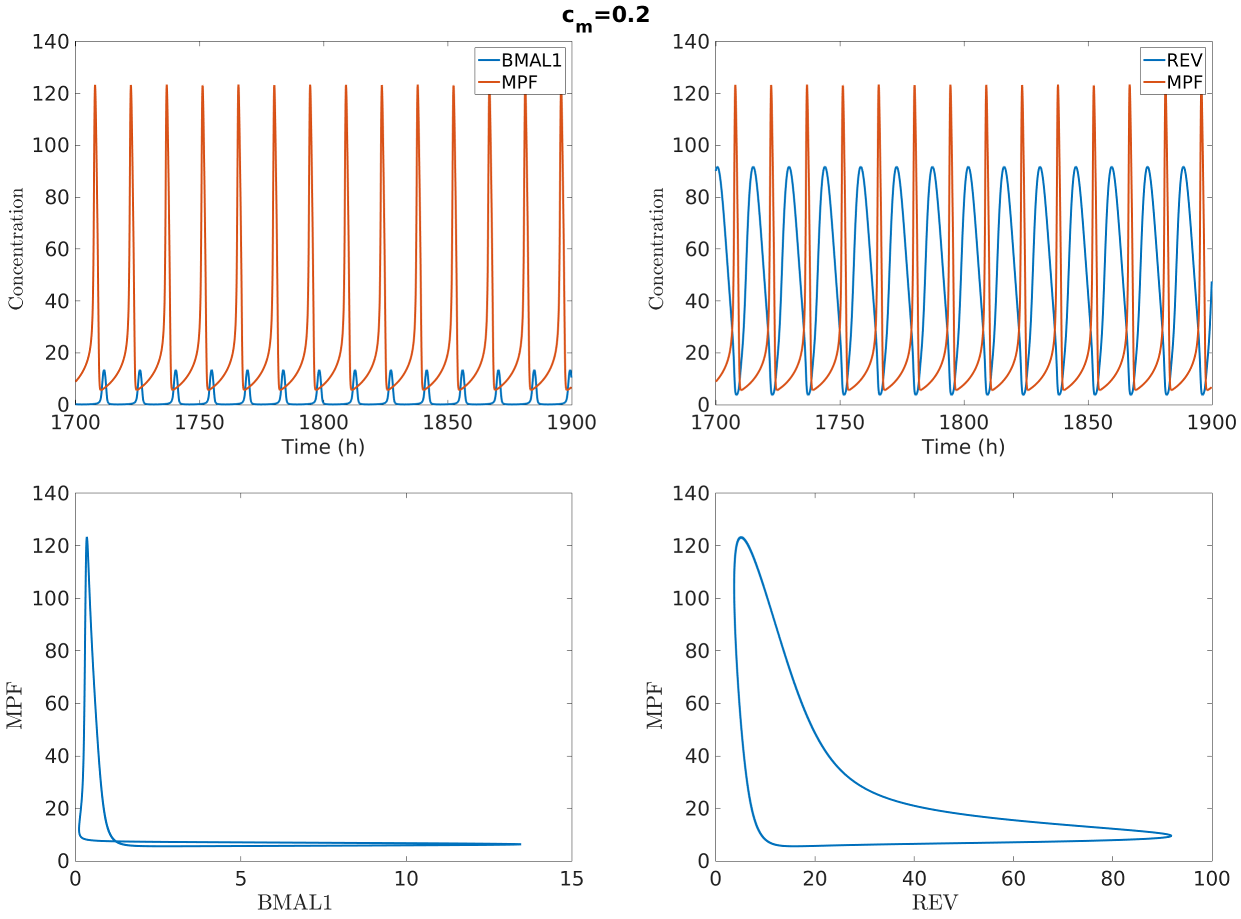

Fig. 4: **Oscillations and phase portraits of BMAL1, REV and MPF in a 1:1 period-lock.** With GF = 40, the coupling strength $c_m = 0.2$ results in a solution with a 1:1 period-lock, where $T_{clock} = T_{cell\ cycle} = 14.5$ h. The system is synchronized and phase-locked.

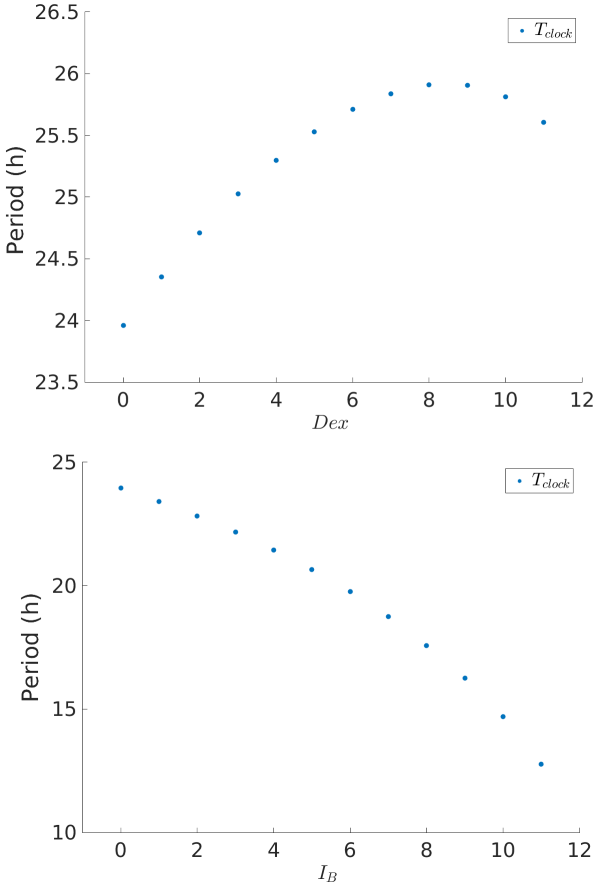

Fig. 5: **Variation of the intrinsic clock period with the Dex and I_B added inputs.** Dex and I_B are varied in the region of oscillation (from 0 to 11): Dex leads to a slower clock, while I_B accelerates the clock.

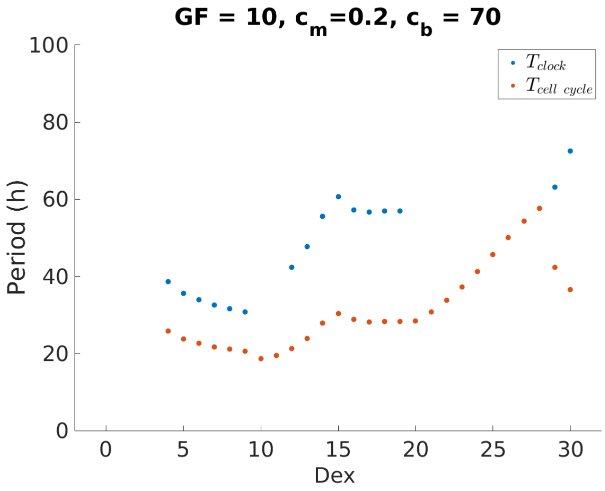

Fig. 6: **Variation of clock and cell cycle periods with Dex in bidirectional coupling.** The clock and cell cycle periods vary non-linearly with Dex: first with a region without oscillation then decreasing and then increasing again.

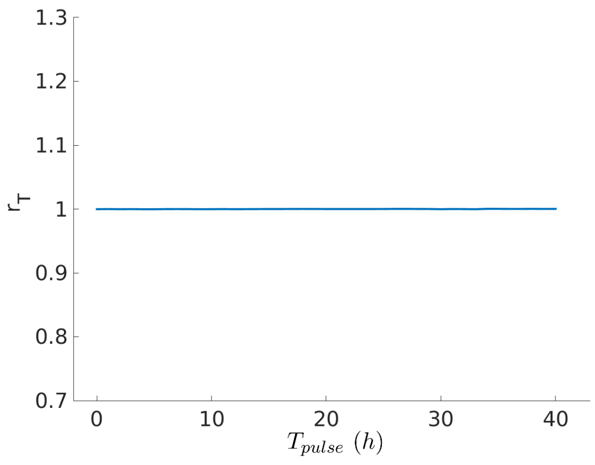

Fig. 7: **Convergence to the 1:1 period-lock state after the application of a Dex-pulse at different circadian phases over the course of two periods.** 1000 hours after the shift caused by the Dex pulse (observed in Fig. 11 of the main article) the system has returned to 1:1 synchronization: changes in period-lock caused by Dex input are transient.

3 References

References

[1] Perko L. Differential equations and dynamical systems. vol. 7. Springer Science & Business Media; 2013.

[2] Feillet C, Krusche P, Tamanini F, Janssens RC, Downey MJ, Martin P, et al. Phase locking and multiple oscillating attractors for the coupled mammalian clock and cell cycle. Proceedings of the National Academy of Sciences of the United States of America. 2014 Jul;111(27):9828–33.
